# Association of Gender, Diagnosis, and Obesity With Retention Rate of Secukinumab in Spondyloarthropathies: Results Form a Multicenter Real-World Study

**DOI:** 10.3389/fmed.2021.815881

**Published:** 2022-01-13

**Authors:** Alicia García-Dorta, Paola León-Suarez, Sonia Peña, Marta Hernández-Díaz, Carlos Rodríguez-Lozano, Enrique González-Dávila, María Vanesa Hernández-Hernández, Federico Díaz-González

**Affiliations:** ^1^Servicio de Reumatología, Hospital Universitario de Canarias, San Cristóbal de La Laguna, Spain; ^2^Servicio de Reumatología, University Hospital of Gran Canaria Dr. Negrin, Las Palmas de Gran Canaria, Spain; ^3^Unidad de Reumatología, Fuerteventura General Hospital Virgen de la Peña, Las Palmas de Gran Canaria, Spain; ^4^Departamento de Estadística e Investigación Operativa, Universidad de La Laguna, La Laguna, Spain; ^5^Departamento de Medicina Interna, Dermatología y Psiquiatría, Universidad de La Laguna, La Laguna, Spain

**Keywords:** spondyloarthritis, secukinumab, gender, obesity, persistence

## Abstract

**Background:** Secukinumab has been shown effective for psoriatic arthritis (PsA) and axial spondylarthritis (AxSpA) in randomized trials. The aim of this study was to analyze baseline patient and disease characteristics associated with a better retention rate of secukinumab under real-world conditions.

**Patients and Methods:** Real-life, prospective multicenter observational study involving 138 patients, 61 PsA and 77 AxSpA, who were analyzed at baseline, 6, 12 months and subsequently every year after starting secukinumab regardless of the line of treatment. Demographics and disease characteristics, measures of activity, secukinumab use, and adverse events were collected. Drug survival was analyzed using Kaplan-Meier curves and factors associated with discontinuation were evaluated using Cox regression. The machine-learning J48 decision tree classifier was also applied.

**Results:** During the 1st year of treatment, 75% of patients persisted with secukinumab, but accrued 71% (*n* = 32) in total losses (*n* = 45). The backward stepwise (Wald) method selected diagnosis, obesity, and gender as relevant variables, the latter when analyzing the interactions. At 1 year of follow-up, the Cox model showed the best retention rate in the groups of AxSpa women (95%, 95% CI 93–97%) and PsA men (89%, 95% CI 84–93%), with the worst retention in PsA women (66%, 95% CI 54–79%). The J48 predicted secukinumab retention with an accuracy of 77.2%. No unexpected safety issues were observed.

**Conclusions:** Secukinumab shows the best retention rate at 1 year of treatment in AxSpA women and in PsA men, independently of factors such as the time of disease evolution, the line of treatment or the initial dose of the drug.

## Introduction

Spondyloarthropathies (SpA) are a heterogeneous group of chronic inflammatory diseases that share a similar genetic basis, pathogenic mechanisms, and clinical expression (1, 2). Psoriatic arthritis (PsA) and axial spondyloarthritis (AxSpA) are the most prevalent SpA (3, 4) and cause musculoskeletal and extra-articular manifestations that have a great impact on patient functionality and quality of life (5).

Tumor necrosis factor inhibitors (TNFi) have resulted in a major change in the management of the SpA (6, 7). However, not all patients with SpA benefit from TNFi, and in many cases those who do show a limited response over time, which is why new therapeutic strategies for the management of these patients have been the subject of intense research. Secukinumab, a fully human monoclonal antibody targeting Interleukin-17A has shown efficacy and safety in the treatment of both active PsA (8, 9) and AxSpA (1, 10) patients in randomized controlled clinical trials (RCTs). Although information from RCTs can provide high-quality information, their strict inclusion criteria and short follow-up periods often limit the applicability of their results to routine clinical practice (11, 12). Therefore, observational studies based on retrospective or prospective data, which rely on actual medication use among real patients, provide more applicable clinical information than RCTs, however.

Retention rates (drug survival) analyzed in the context of observational studies provide key information on the performance of biologics in terms of their effectiveness, safety, compliance, and convenience of use under real-world conditions. The retention of secukinumab has been analyzed in retrospective observational series and registries focused on safety and effectiveness (13–15), even in comparison with TNFi (16–18). However, knowledge about the influence that patient characteristics, diagnosis, or drug use (dose or line of treatment) might have on secukinumab persistence in patients with PsA and AxSpA under real-life conditions is still scarce.

In this work, we have studied secukinumab retention in patients with PsA and AxSpA by analyzing baseline patient and disease characteristics that are associated with improved drug survival in routine clinical practice. This study may aid in identifying the subgroups of SpA patients who are most likely to enjoy a higher retention rate with secukinumab and, consequently, most benefit from this therapy.

## Patients and Methods

### Study Design and Data Sources

This is an observational study based on a prospectively recorded database of patients with SpA treated with secukinumab. PsA and AxSpA patients were identified in the biologics databases maintained by three Hospitals in the Canary Islands (Atlantic islands 28° N, 16° W), Spain: Hospital Universitario de Canarias, Hospital Universitario Doctor Negrín and Hospital General de Fuerteventura Virgen de la Peña. From each database, demographic patient characteristics (age, gender, diagnostic, clinical presentation as axial, peripheral or mixed, disease evolution time, concomitant medication, including conventional synthetic (cs) disease-modifying antirheumatic drugs (DMARDs) or non-steroidal anti-inflammatory drugs (NSAIDs), body mass index (BMI), start and stop dates and dose of secukinumab, together with previous biologics and disease activity measures, were retrieved. Due to an expected difference in the treatment retention and response between naïve and previously treated with one or more biologic (b) DMARD patients, information was also collected by the first, second and third (or more) of secukinumab line of treatment. History of previous TNFi use was also collected.

### Patients and Follow-Up

Patients in the databases (*n* = 138) diagnosed with AxSpA by ASAS (Assessment of Spondyloarthritis International Society) classification criteria (19) (*n* = 77) or PsA by CASPAR (ClASsification criteria for Psoriatic Arthritis) (20) (*n* = 61), and who started secukinumab from November 2015 to September 2020 and who persisted with the treatment for more than 3 months were included. Follow-up started at the start date of secukinumab administration and ended at the stop date for the treatment, death, or the end of the study (31 December 2020), whichever occurred first.

### Treatment Response

Treatment response was evaluated at 6 and 12 months after start, and then afterward every year. Simple activity indices such as the number of tender joints (NTJ), swollen joints (NSJ), erythrocyte sedimentation rate (ESR) and C-reactive protein (CRP) were collected. In PsA patients, disease activity was assessed by DAS-ESR (Disease Activity Score 66/68) (21) and in AxSpA patients by ASDAS-CRP (Ankylosing Spondylitis Disease Activity Score) (22) and BASDAI (Bath Ankylosing Spondylitis Disease Activity Index) (23), data that was collected during different study visits. HAQ (Health Assessment Questionnaire) was also assessed in all participating patients.

### Treatment Retention

**Figure 1:**
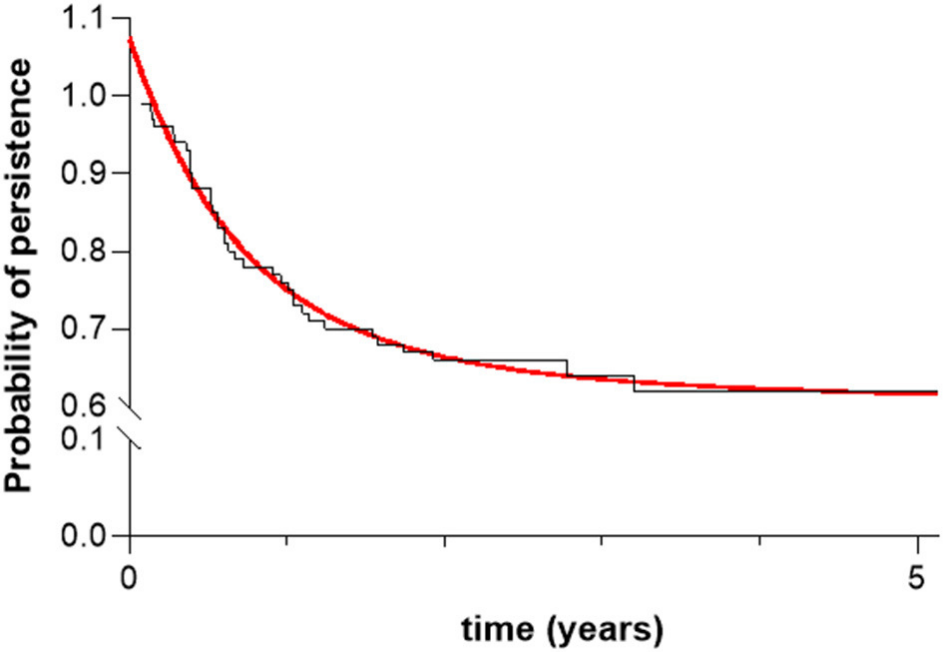
Survival curve of secukinumab in the entire population. Red line represents the fit of the Kaplan-Meier survival curve to the exponential two-phase decay equation model (*R*^2^ = 0.98).

The overall retention of secukinumab was defined as the probability of long-term drug survival of up to 5 years of treatment, as described by Kaplan–Meier curves. The observed survival curve of secukinumab was fitted to a two-phase exponential decay curve (*R*^2^ = 0.98) (Figure 1) (24). The change in the trend of the curve from fast to slow decay was calculated according to the following formula:


$$\begin{array}{l} {Retention\;}_{0}-SpanFast\\ =\;{Retention\;}_{0}\;\left({Retention\;}_{0}\;-\;Plateau\right)\;*PercentFast\;*\;0.01; \end{array}$$

where Retention _0_ is the survival value when X(time) is zero, Plateau is the survival value at infinite times = 0.6095 and PercentFast, 75.06%, is the fraction of the span (from Retention_0_ to Plateau) where the decay is fastest. Appling this formula, the retention rate when the trend changed from fast to slow decay was 0.7250, corresponding to 1.05 years of follow-up in the observed values.

Since long-term retention of secukinumab was strongly conditioned by its survival at the end of the 1st year, factors associated with drug retention were analyzed during that period. Hazard ratio (HR) of discontinuation during the 1st year of treatment was assessed through Cox proportional hazard regression models as adjusted for age (continuous in years), gender, BMI (categorized as obesity ≥30 kg/m^2^, or <30 kg/m^2^), diagnosis (categorical), disease evolution time (continuous in years), line of treatment (categorized as first, second or higher lines) and initial secukinumab dose (categorical) as the variable of interest. The logistic regression model was constructed using the backward Wald variable selection method and *p*-values for entry and removal of variables of *p* < 0.05 and *p* < 0.10, respectively. The backward stepwise (Wald) method selected diagnosis and obesity as relevant variables, while gender was included as a fixed factor due to its impact on the interaction analyses. These variables and the interactions between obesity^*^gender and diagnosis^*^gender were also analyzed using a multivariable Cox proportional hazards regression model during the 1st year of treatment. Dispersion measures of the Cox model were expressed as standard error or 95% confidence interval (95%CI). Patients were censored at the time they switched to another biologic, either by inefficacy or adverse events, at the end of the study period (31 December 2020) or 1 year from start of treatment.

For exploratory purpose, variables included in the logistic regression model were also analyzed using the machine-learning classifier J48 (25). In this analysis the outcome was retention at 1 year of treatment and all patients who reached at least 1 year of follow-up were included, regardless of whether or not they continued to take secukinumab at that time (*n* = 123). Since the sample was unbalanced in favor of retention after 1 year, before applying the J48 classification tree [version of C4.5 in Weka, Ross Quinlan (1993) C4.5: Programs for Machine Learning. Morgan Kaufmann Publishers, San Mateo, CA.], the supervised filter ClassBalancer was used, in order to provide to both categories: retention or non-retention, equal probability of occurrence.

The discrimination property of this model was assessed with the area under the receiver operating characteristic curve (AUROC). The internal validation of the machine-learning models was performed with Weka 3.8.0 (Waikato Environment for Knowledge Analysis, GNU-GPL) using a 10-fold cross-validation method (26).

All statistical analyses were performed using SPSS (version 25, IBM SPSS, Armonk, NY) and Weka 3.8.0 software. Results were considered significant if *p* < 0.05.

### Statistics

Data are summarized as relative frequencies for categorical variables, means ± standard deviation for normally distributed variables and median (interquartile range IQR P25; P75) for non-normal data. Comparisons were performed using the Pearson's chi-square test, Kruskal-Wallis test, or Mann Whitney *U*-test and the ANOVA or t-student according to the type of variable and the number of groups to be compared. For the longitudinal study, a mixed effects linear model with a random intercept and slope was applied to include patients with different follow-up periods. The evaluation of factor interactions over time allowed for the calculation of reduction rates for the variables analyzed. Survival analysis of secukinumab was performed using a Cox regression model adjusting for gender, initial dose, time of disease progression, line of treatment and diagnosis of rheumatic disease.

## Results

A total of 138 patients were included in the analysis, 77 AxSpA and 61 PsA with a mean age at start of secukinumab of 47 ± 10.3 years with no significant differences between AxSpa and PsA patients (Table 1). Ninety patients were men (65%), 57 (74%) AxSpA and 33 (54%) PsA (*p* = 0.019). With respect to BMI, 17 (22%) AxSpA and 19 (31%) PsA patients were within a range of obesity of ≥ 30 kg/m^2^ (*p* = 0.329). Most AxSpA patients started secukinumab at 150 mg (88%) in monotherapy (*n* = 56, 73%), whereas PsA patients initiated secukinumab at 150 or 300 mg in similar proportions, although this was preferentially associated with a csDMARDs (*n* = 40, 66%). Secukinumab was used as the first bDMARD in 47 (34%) patients, as a second option in 27 (20%) and as a third or higher option in 63 (46%) with no significant differences in terms of diagnosis (*p* = 0.5). Table 1 shows a description of other demographic and disease characteristics, as well as clinical and biological activity of the study population at start of secukinumab, both by diagnosis and in total.

**Table 1:** Baseline demographic and clinical characteristics; secukinumab use in the study population.

	**AxSpA (*n =* 77)**	**PsA (*n =* 61)**	***p*-valor**	**Total (*n =* 138)**
Age (years), mean ± SD	47.4 ± 11.0	47.6 ± 9.4	0.931	47.5 ± 10.3
Male, n (%)	57 (74)	33 (54)	0.019	90 (65)
Disease duration (years), median (IQR)	12.9 (5.7–23.3)	7.8 (3.6–14.7)	0.001	9.4 (4.8–18.9)
BMI kg/m^2^, mean ± SD	26.7 ± 4.2	28.1 ± 5.0	0.066	27.3 ± 4.6
≥30, n (%)	17 (22)	19 (31)	0.329	36 (26)
Joint involvement, n (%)			<0.001	
Axial radiographic	67 (87)	1 (2)		68 (49)
Axial non-radiographic	10 (13)	-		10 (7)
Peripheral	-	38 (62)		38 (27)
Mixed	-	22 (36)		22 (16)
**Clinical activity, median (IQR)**				
NTJ	1 (0–6)	3 (1–8)	<0.001	2 (0–8)
NSJ	0 (0–1)	1 (0–3)	0.003	0 (0–2)
**Disease activity indexes, mean** **±** **SD**				
DAS-ESR	-	3.8 ± 1.4		
ASDAS-CRP	3.7 ± 0.7	3.6 ± 0.8	0.506	3.5 ± 0.7
BASDAI	6.5 ± 1.6	6.2 ± 2.3	0.674	6.4 ± 1.7
BASFI	6.0 ± 2.3	5.3 ± 2.5	0.366	5.9 ± 2.3
**Biologic activity, median (IQR)**				
ESR mm/h	7.5 (3–10.2)	11.5 (4.7–27)	0.003	9 (3–18.7)
CRP mg/L	4.5 (1.2–10.5)	5.6 (1.7–12.6)	0.263	4.9 (1.5–11.4)
HLAB27 +, %, (n+/n done)	95 (59/62)	21 (7/33)	<0.001	69 (66/95)
HAQ	-	1.3 ± 0.7		-
Initial dose of Secukinumab, n (%)			0.001	
150 mg	68 (88)	28 (46)		96 (70)
300 mg	9 (12)	33 (54)		42 (30)
Secukinumab in monotherapy, n (%)	56 (73)	21 (34)	0.001	77 (56)
Líne of Secukinumab, n (%)			0.501	
1	29 (38)	18 (30)		47 (34)
2	13 (17)	14 (23)		27 (20)
≥3	34 (45)	29 (48)		63 (46)
Previous TNFi, n (%)	47 (61)	41(68)	0.473	88 (64)

*AxSpA, Spondyloarthritis; ASDAS, ankylosing spondylitis disease activity score; BASDAI, Bath ankylosing spondylitis disease activity index; BASFI, Bath ankylosing spondylitis functional index; CRP, C-reactive protein; DAS, disease activity index; ESR, erythrocyte sedimentation rate; HAQ, Health assessment quality; IQR, interquartile range; NSJ, number of swollen joints; NTJ, number of tender joints; PsA, Psoriatic arthritis; SD, standard deviation; TNFi, TNF inhibitor*.

Supplementary Tables 1, 2 show demographic characteristics, joint involvement, disease, and biological activity, as well as secukinumab use by lines of treatment in PsA and AxSpA patients, respectively. Overall, there was a significant trend toward using 300 mg as a starting dose as the treatment line progressed, a pattern that was more marked in PsA patients (*p* < 0.001) than in AxSpA patients (*p* = 0.038).

### Effectiveness

An overall and sustained reduction in disease activity indices (Table 2 and Supplementary Figure 1) was observed from the baseline visit to 3 or more years of follow-up. Peripheral arthritis in PsA patients, as assessed by the DAS-ESR, improved significantly from a mean of 3.8 ± 1.4 to 2.5 ± 0.5 (*p* < 0.001) (Table 2 and Supplementary Figure 1A).

**Table 2:** Variation of disease activity during treatment with secukinumab.

	**Baseline (*n =* 138)**	**6 months (*n =* 115)**	**1 year (*n =* 79)**	**2 years (*n =* 52)**	**≥3 years (*n =* 24)**	***p*-valor**
**Monotherapy**, n (%)	77 (55.8)	75 (65.2)	56 (71)	39 (75)	20 (83)	<0.001
**Disease activity**						
**PsA**						
ESR, median (IQR)	11.5 (4.8–27)	7 (3–14)	7 (5–9.3)	5.5 (2.7–13.2)	7 (4.2–26)	0.090
CRP, median (IQR)	5.6 (1.8–12.7)	2.8 (1.5–7.9)	3.6 (2.1–6.8)	3.5 (1.1–7.4)	7.7 (2.5–15.1)	0.402
DAS-ESR, mean ± SD	3.8 ± 1.4	2.7 ± 1.6	2.8 ±1.3	2.2 ± 1.2	2.5 ± 0.5	<0.001
**AxSpA**						
ESR, median (IQR)	7.5 (3–10.2)	6 (2–10)	3 (2–6.2)	5.0 (2.7–7.2)	4 (2–5.2)	0.031
CRP, median (IQR)	4.6 (1.3–10.5)	3.2 (1.1–6.7)	3.4 (1.4–7.7)	1.4 (0.8–4.2)	1.6 (0.9–2.5)	0.082
ASDAS-CRP, mean ± SD	3.4 ± 0.8	2.6 ± 1	2.5 ± 0.8	2.1 ± 1.2	2.1 ± 1.0	<0.001
BASDAI, mean ± SD	6.5 ± 1.6	5.0 ± 2.5	4.6 ± 2.3	4.2 ± 2.2	4.0 ± 2.5	<0.001
BASFI, mean ± SD	6.0 ± 2.3	4.8 ± 2.6	4.2 ± 2.4	4.1 ± 2.8	3.8 ± 2.6	0.001
**HAQ**, mean ± SD	1.4 ± 0.7	0.9 ± 0.8	0.8 ± 0.8	0.7 ± 0.6	0.9 ± 0.8	0.937

*AxSpA, Spondyloarthritis; ASDAS, ankylosing spondylitis disease activity score; BASDAI, Bath ankylosing spondylitis disease activity index; BASFI, Bath ankylosing spondylitis functional index; CRP, C-reactive protein; DAS, disease activity index; ESR, erythrocyte sedimentation rate; HAQ, Health assessment quality; IQR, interquartile range; PsA, Psoriatic arthritis; SD, standard deviation*.

In patients with axial involvement, there was a statistically significant improvement in the ASDAS-CRP, from a mean of 3.4 ± 0.8 to 2.1 ± 1.0 (*p* < 0.001) (Table 2 and Supplementary Figure 1B), in BASDAI score from a mean of 6.5 ± 1.6 to 4.0 ± 2.5 (*p* < 0.001), and in the BASFI score from a mean of 6 ± 2.3 to 3.8 ± 2.6 (*p* = 0.001) (Table 2).

### Treatment Retention

According to the Kaplan-Meier curve (Figure 1), the estimated annual retention rate of secukinumab in the entire population was, consecutively, 75, 66, 64, 62, and 62% during the 5 years analyzed. The estimated global mean of secukinumab survival was 22.3 ± 15.6 months, very similar in both pathologies: 21.9 ± 15.4 months for AxSpA and 22.9 ± 15.9 for PsA patients. When drug survival was estimated by Kaplan-Meier curves independently in AxSpA and PsA patients, 70 and 55% persisted with secukinumab at 5 years, respectively, with no significant differences noted (*p* = 0.236 by log-rank test).

As stated in the Patients and Methods section, the Kaplan-Meier curve was fitted to a two-phase exponential decay curve (*R*^2^ = 0.98) (Figure 1), with the rapid decline occurring during the 1st year of treatment, a period in which 71% (*n* = 32) of the total losses (*n* = 45) of secukinumab took place. Since the long-term survival of secukinumab was strongly conditioned by the retention rate recorded at the end of the 1st year, we analyzed factors associated with drug survival during that period. Such factors were identified using multivariable Cox regression analysis using a backward stepwise (Wald) method. The variables of diagnosis, obesity and gender were selected as relevant, while age, disease evolution time, line of treatment, and initial secukinumab dose were excluded (see Patients and Methods section). Table 3 shows the HRs for discontinuation of selected factors and their interactions. After 1 year of treatment, patients diagnosed with PsA were less likely to persist with secukinumab than those with AxSpA with an HR of 12.32 (95% CI 1.6–94.9, *p* = 0.016), obese patients showed a non-significant trend to discontinuation with respect to the non-obese with an HR of 2.54 (95% CI 0.99–6.50, *p* = 0.051) and women showed a trend toward a better retention rate that men with an HR of 0.29 (95% CI 0.04–2.28, *p* = 0.241). Analyses of interactions between factors showed that obese women had a higher probability of retention at 1 year of treatment with secukinumab, with an HR of 0.046 (95% CI 0.005–0.44, *p* = 0.007), while in PsA men HR measured 0.03 (95% CI 0.003–0.31, *p* = 0.003). Table 4 shows the predicted retention rate of secukinumab by the Cox model at 1 year of follow-up according to diagnosis, gender, and the presence or absence of obesity. The best secukinumab retention rate was observed in the group of AxSpa women with a 95% (95% IC 93–97%) and in PsA men with an 85% (95% IC 84–93%), while the worst was in PsA women with 66% (95% IC 54–79%), results consistent with those obtained in the direct analysis of the sample (Supplementary Table 3).

**Table 3:** Multivariate Cox regression of discontinuation after 1 year of Secukinumab treatment in the entire population.

	**β**	**SE**	**Wald**	***p*-value**	**HR (95%CI)**
Diagnosis (Ref. AxSpA)	2.511	1.042	5.812	0.016	12.32 (1.60–94.9)
Obesity (Ref. No)	0.933	0.479	3.795	0.051	2.54 (0.99–6.50)
Gender (Ref. Male)	−1.229	1.047	1.377	0.241	0.29 (0.04–2.28)
**Interactions**					
Obesity*gender (Ref. No woman obese)	−3.075	1.147	7.193	0.007	0.046 (0.005–0.44)
Diagnosis*gender (Ref. No man PsA)	−3.508	1.186	8.752	0.003	0.03 (0.003–0.31)

*AxSpA, Spondyloarthritis; PsA, Psoriatic arthritis; SE, standard error*.

**Table 4:** Estimated retention rate during the 1st year of secukinumab treatment according to diagnosis, gender, and BMI.

**Diagnosis**	**Retention %**	**95%CI**	**Gender**	**Retention %**	**95%CI**	**BMI**	**Retention %**	**95% CI**
AxSpA	82%	(74%; 89%)	Female	95%	(93%; 97%)	<30 kg/m^2^	93%	(89%; 96%)
						≥30 kg/m^2^	99%	(98%; 100%)
			Male	77%	(68%; 86%)	<30 kg/m^2^	80%	(72%; 89%)
						≥30 kg/m^2^	64%	(50%; 78%)
PsA	78%	(70%; 87%)	Female	66%	(54%; 79%)	<30 kg/m^2^	57%	(42%; 73%)
						≥30 kg/m^2^	91%	(87%; 95%)
			Male	89%	(84%; 93%)	<30 kg/m^2^	91%	(88%; 96%)
						≥30 kg/m^2^	81%	(73%; 89%)

*AxSpA, Spondyloarthritis; BMI, Body mass index; 95% CI, 95% Confidence interval; PsA, Psoriatic arthritis*.

**Figure 2:**
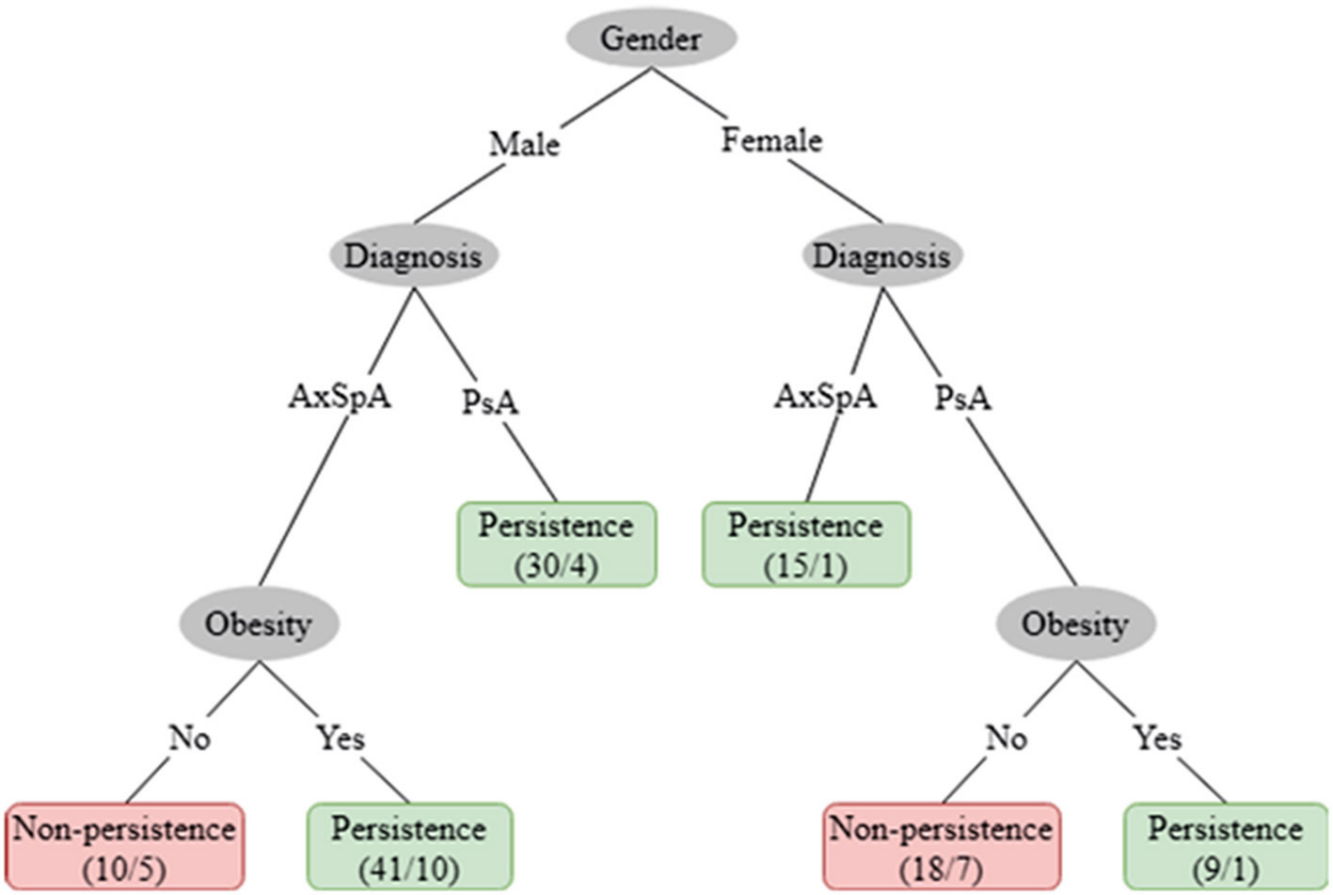
Decision tree J48. The figures in the boxes represent the number of patients that the algorithm included in each branch, divided by the number of patients that the algorithm did not correctly predict persistence (green boxes) or non-persistence (red boxes) after 1 year of treatment.

For exploratory purposes, Figure 2 provides a representation of the J48 classification tree. The root classifier was gender, with diagnosis and obesity representing the branches of the algorithm. The positive predictive value of the classification was 83.1% with a negative predictive value of 57.1% and an area under the ROC curve of 0.743 (*p* < 0.001). The accuracy of the model was 77.4% (95/123).

### Reasons for Discontinuation and Safety

Of the 45 patients who discontinued secukinumab treatment at the end of follow-up, 38 (84%) stopped because of ineffectiveness, 4 (9%) from side effects (Table 5), 2 by dermal toxicity and 2 by recurrent urinary tract infections, and 3 (7%) due to other causes such as gestational desire, diagnosis of inflammatory disease, loss to follow-up and exitus (one patient for each). During the 1st year, the main reason for discontinuation was inefficacy (84%, *n* = 27).

**Table 5:** Adverse events collected.

	**6 months (*n =* 115)**	**1 year (*n =* 79)**	**2 years (*n =* 52)**	**≥3 years (*n =* 24)**	***p*-value**
Adverse events. n (%)					0.013
Infections (upper tract/genito-urinary tract/abdominal/fungal)	8 (7)	16 (20)	10 (19)	3 (12)	
Toxicity	0	2 (2)	0	0	
Alteration of stool habit	2 (2)	0	0	0	
Psychiatric disturbance	1 (1)	0	0	0	

## Discussion

The most important findings of this work can be summarized as follows: (1) in patients with SpA, the retention rate of secukinumab after a year of treatment under real-world conditions was 75%; (2) at that time, the best retention rate of secukinumab was recorded in AxSpA women and in PsA men, independently of factors such as time of disease evolution, line of treatment or the initial dose of the drug; (3) obesity was not a factor associated with poor retention rates of secukinumab in our series; and (4) under real world conditions, secukinumab shows a effectiveness and safety profile equivalent to those published in pivotal RCTs in SpA patients.

The retention rate indicates the probability of persisting with the same drug over time and it is a marker of overall treatment efficacy, safety, and patient compliance (27). Long-term retention is key to obtaining the greatest benefit from biologic treatments. Consequently, it is important to consider the information provided by both open-label extension phases of RCTs and, preferably, studies under routine clinical practice conditions in order to identify patient and disease characteristics potentially associated with better survival rates of these compounds. In open-label extensions of clinical trials with secukinumab, involving both biologic naïve patients and those who had failed TNFi, 84% of AxSpa patients (28) and 64% of PsA patients (29) remained on the drug after 5 years of follow-up, a retention rate higher than those obtained in our real-world study (70 and 55%, respectively), an expected finding, since the retention rate tends to be higher in clinical trials than in real-life studies due to the strict inclusion/exclusion criteria and the close follow-up of patients in this first scenario. To our knowledge, no studies with secukinumab that reached 5 years of follow-up under clinical practice conditions have been reported, as our study now does.

When analyzing the retention rate of biologics in patients with rheumatic diseases in real-life settings, the loss of retention of these drugs tends to be most accelerated during the first 12–18 months of treatment initiation, with a trend toward a more stable persistency thereafter (13–15). In our study, secukinumab showed similar behavior, with a loss recorded in 34 patients during the 1st year, 71% of the total losses observed during the 5-year follow-up. By diseases, the sample data showed in our study a retention rate of secukinumab at the 1st year of treatment in AxSpa and PsA of 78 and 73%, respectively. This is in the range of what has been published in similar real-life studies, which reported overall retention rates of 76 and 66%, respectively (13–15). Also accordingly to previous studies (13–15), the main cause of secukinumab discontinuation in our series was inefficacy (84%).

The assessment of baseline patient and disease characteristics that are associated with higher retention of the biologic may aid in identifying the most appropriate patient profile for achieving better long-term efficacy and safety. The main objective of this work was to identify the patient profile associated with a better secukinumab survival. Since, according to our results, the long-term survival of secukinumab was conditioned by the retention rate in the 1st year, we analyzed factors associated with drug survival during that initial treatment period. In the multivariate Cox regression analysis model, the backward stepwise method (Wald) selected diagnosis, obesity, and gender as relevant variables, excluding age, time of disease progression, line of treatment, and initial secukinumab dose. Although a lower secukinumab retention rate has been reported in SpA patients previously treated with biologics (13, 14), other real-life studies have shown that the secukinumab retention rate at 12 months was not influenced by the line of treatment in patients with AxSpa (15, 30) or PsA (15, 31), nor by the dose used (30), which is consistent with our own study. In our study, retention of secukinumab was not associated with time, diagnosis or patient age, results that have similarly been reported by other studies (13, 14).

In the multivariable analysis, PsA patients were less likely to retain secukinumab than AxSpA patients, with obesity and gender showing no statistical significance in terms of drug retention after 1 year of treatment. However, when interactions were analyzed, obesity and gender proved to be very relevant factors in secukinumab retention, with a higher probability of retention in obese women and in men with PsA. When retention rates at 1 year of secukinumab treatment were analyzed using the Cox model, the best retention rate was observed in the group of AxSpa women at 95% and in PsA men at 85%, while the worst rate was in the women PsA group at 66%. These results were consistent with those obtained in the direct analysis of the sample. Previous real-life studies analyzing secukinumab persistence in patients with AxSpa and PsA did not report consistent differences in retention rates based on diagnosis and gender (14, 15). One possible explanation for this is that these previous studies did not perform multivariate interaction analysis as was done in our work. In fact, in our study gender and obesity did not reach statistical significance in the multivariate analysis but showed a very important effect when included in the interaction study.

In an exploratory manner we utilized machine learning (ML) algorithms that can make reasonably accurate decisions when provided with relevant data. J48 (C4.5) is the most widely used decision-tree algorithm, also known as a statistical classifier. J48 can handle both numerical and categorical data (32), is easy to implement, and deals with both noise and missing values (33). However, the performance of J48 is not good for a small data set (33). In our study, J48 showed a positive predictive value of 83.1%, but a negative predictive value of only 57.1%, with an overall accuracy of 77.1%, only 2 points above the survival of secukinumab shown in the sample under real-life conditions.

Obesity is a prevalent comorbidity in different inflammatory joint diseases (34), with particular relevance in PsA patients (35). Biologics may influence the weight and body composition of treated patients (36), while obesity, in turn, may influence the clinical response to these agents (37). The clinical response to TNFi is attenuated by obesity, an effect that is less evident with IL-6 inhibitors and rituximab, and negligible with abatacept [reviewed (38)]. While secukinumab data on its efficacy in patients with SpA based on body weight are scarce, its efficacy does not appear to be dependent upon patient BMI in PsA (15, 39) or AxSpA (15) patients. In our series, obesity was not a factor associated with a poor retention rate of secukinumab; in fact, it may even be a predictor of longer secukinumab retention in women. Although we have not found an obvious explanation for this differential finding between genders in persistence to secukinumab in obese, recently, Alonso et at. have described a positive association of persistence and obesity in a series of 59 patients with PsA treated with secukinumab (14), but unfortunately in this study a gender^*^obesity interaction analysis was not performed. Such findings may aid physicians in therapeutic choices for obese/overweight patients by prioritizing secukinumab over anti-TNFs in both obese AxSpA and PsA patients.

The safety profile of secukinumab in our real-life setting study was not much different to what has been previously reported in RCTs and their long-term extension studies (9, 40, 41).

Our study has some limitations, firstly, the sample size was relatively small, and the study was performed in three hospitals located in the Canary Islands. While genetic studies of the current population of the Canary Islands have shown that 90% of the paternal lineage is of European origin (42), the results of this study may not be generalizable to other populations. Secondly, this is a retrospective study and although prospective databases were used, retrospective data collection can entail a certain risk of bias due to the lack of standardization. Also, another potential limitation of this study is that factors such as smoking status, intensity of dermal involvement or level of joint damage were not considered in the analyses.

In conclusion, in this real-world study secukinumab demonstrated a 75% retention rate at 1 year of treatment with an adequate safety profile in patients with SpA. This study identifies women with AxSpA and men with PsA as the patient groups with a better secukinumab retention rate of around 90% at 1 year of treatment, independent of factors such as time to disease progression, line of treatment or initial drug dose. Interestingly, obesity was not associated with a lower secukinumab retention rate in our series.

## Data Availability Statement

The raw data supporting the conclusions of this article will be made available by the authors, without undue reservation.

## Ethics Statement

The studies involving human participants were reviewed and approved by the Ethics Committee of the Hospital Universitario de Canarias, Spain (SECUREAL Study 03/2021). Written informed consent for participation was not required for this study in accordance with the national legislation and the institutional requirements.

## Author Contributions

AG-D, PL-S, SP, and MH-D: data collection, analysis, and verification. MH-H: study design, data collection, and writing. EG-D: statistical analysis and data interpretation. CR-L and FD-G: study design, data interpretation, and writing. All authors read, corrected, and approved the final version submitted.

## Funding

This work was supported by the Asociación para la Ayuda a la Investigación del Hospital Universitario de Canarias (REUNINVES).

## Conflict of Interest

The authors declare that the research was conducted in the absence of any commercial or financial relationships that could be construed as a potential conflict of interest.

## Publisher's Note

All claims expressed in this article are solely those of the authors and do not necessarily represent those of their affiliated organizations, or those of the publisher, the editors and the reviewers. Any product that may be evaluated in this article, or claim that may be made by its manufacturer, is not guaranteed or endorsed by the publisher.

## Supplementary Material

Supplementary Figure 1 Variations in activity indices over time. **(A)** Change from baseline in DAS-ESR (DDAS-ESR) and **(B)** in ASDAS-PCR (DASDAS-PCR) in patients treated with secukinumab.

Supplementary Table 1 Baseline demographic and clinical characteristics of PsA patients by line of Secukinumab treatment.

Supplementary Table 2 Baseline demographic and clinical characteristics of AxSpA patients by line of Secukinumab treatment.

Supplementary Table 3 Sample retention rate during the first year of secukinumab treatment according to diagnosis, gender, and BMI.
