# Supplementary material for: Association of Gender, Diagnosis, and Obesity With Retention Rate of Secukinumab in Spondyloarthropathies: Results Form a Multicenter Real-World Study
Source: Front Med (Lausanne). 2022 Jan 13;8:815881. doi: 10.3389/fmed.2021.815881 (PMC8792854; doi:10.3389/fmed.2021.815881)
Supplement: Supplementary Figure 1 — Variations in activity indices over time. (A) Change from baseline in DAS-ESR (DDAS-ESR) and (B) in ASDAS-PCR (DASDAS-PCR) in patients treated with secukinumab. [file Data_Sheet_1.docx]

Supplementary Material

# Supplementary Tables

**Supplementary Table 1**. Baseline demographic and clinical characteristics of PsA patients by line of Secukinumab treatment.

|  | First-line (n=18) | Second-line (n=14) | ≥Third line  (n= 29) | p-valor | |
| --- | --- | --- | --- | --- | --- |
| Age (years), mean±SD | 46.6 ± 7.5 | 46.8 ± 11.2 | 48.7 ± 9.9 | 0.713 | |
| Male, n (%) | 11 (61.1) | 6 (42.9) | 16 (55.2) | 0.582 | |
| Disease duration (years), median (IQR) | 2.8 (1.4-9.8) | 6.9 (3.8-8.7) | 11.4 (6.8-17.0) | 0.002 | |
| BMI kg/m^2^, mean±SD | 28.6 ± 5.2 | 27.0 ± 4.2 | 28.4 ± 5.4 | 0.615 | |
| ≥30, n (%) | 8 (44) | 3 (21) | 8 (28) | 0.321 | |
| Joint involvement, n (%) |  |  |  | 0.040 | |
| Peripheral | 15 (83.3) | 7 (50) | 16 (55.2) |  | |
| Mixed | 2 (17.7) | 7 (50) | 13 (44.8) |  | |
| Clinical activity, median (IQR) |  |  |  |  | |
| NTJ | 1 (0-6) | 2 (1-9) | 3 (1-10) | 0.358 | |
| NSJ | 1 (0-3) | 0 (0-1.5) | 0 (0-2) | 0.509 | |
| DAS-ESR, mean±SD | 3.68 ± 1.47 | 3.55 ± 1.25 | 3.95 ± 1.55 | 0.735 | |
| Biologic activity, median (IQR) |  |  |  |  | |
| ESR mm/h | 12 (7-27.5) | 8 (2.7-25.7) | 14 (4-27) | 0.570 | |
| CRP mg/L | 6.4 (3.3-25.5) | 4.1 (1.9-9.2) | 5.5 (0.7-10.1) | 0.116 | |
| HAQ, media ± DE | 1.47 ± 0.71 | 1.14 ± 0.42 | 1.36 ± 0.83 | 0.628 | |
| Line of Secukinumab, n (%) |  |  |  | <0.001 | |
| 150 mg | 16 (88.9) | 6 (42.9) | 6 (20.7) |  | |
| 300 mg | 2 (11.1) | 8 (57.1) | 23 (79.3) |  | |
| Secukinumab in monoterapia, n (%) | 6 (33.3) | 6 (42.9) | 9 (31.0) | 0.742 | |
| Previous TNFi, n % | - | 12(85.7) | 29(100) | <0.001 | |
| Dose intensification, n (%) | 5 (33.3) | 1(7.1) | 4 (13.8) | 0.257 | |
| CRP: C-reactive protein; DAS: disease activity index; ESR: erythrocyte sedimentation rate; HAQ: Health assessment quality; IQR: interquartile range; NSJ: number of swollen joints; NTJ: number of tender joints; PsA: Psoriatic arthritis; SD: standard deviation; TNFi: TNF inhibitor. | | | | |  |

**Supplementary Table 2**. Baseline demographic and clinical characteristics of AxSpA patients by line of Secukinumab treatment.

|  | First-line (n=29) | Second-line (n=13) | ≥Third line  (n= 34) | p-valor |
| --- | --- | --- | --- | --- |
| Age (years), mean±SD | 45.1 ± 10.2 | 48.6 ± 7.4 | 49.7 ± 12.3 | 0.238 |
| Male, n (%) | 22 (75.9) | 11 (84.6) | 24 (70.6) | 0.605 |
| Disease duration (years), median (IQR) | 9.0 (4.5-20.3) | 7.6 (3.9-17.3) | 20.8 (9.5-29.3) | 0.005 |
| BMI kg/m^2^, mean±SD | 26.3 ± 3.8 | 27.9 ± 4.4 | 26.7 ± 4.4 | 0.530 |
| ≥30, n (%) | 5 (18) | 3 (23) | 9 (27) | 0.722 |
| Joint involvement, n (%) |  |  |  | 0.028 |
| Axial radiographic | 25 (86.2) | 9 (69.2) | 33 (97) |  |
| Axial non radiographic | 4 (13.8) | 4 (30.8) | 1 (3) |  |
| Disease activity, mean±SD |  |  |  |  |
| ASDAS-CRP | 3.6 ± 0.62 | 3.07 ± 0.73 | 3.48 ± 0.87 | 0.148 |
| BASDAI | 6.38 ± 1.66 | 6.65 ± 1.77 | 6.36 ± 1.84 | 0.844 |
| Biologic activity, median (IQR) |  |  |  |  |
| ESR mm/h | 8 (3-10.5) | 6 (3.2-9.5) | 6 (2-12.2) | 0.932 |
| CRP mg/L | 7.1 (3.2-18.6) | 1.6 (0.8-4.5) | 3.5 (1.0-7.7) | 0.011 |
| Initial dose of Secukinumab, n (%) |  |  |  | 0.038 |
| 150 mg | 29 (100) | 10 (77) | 28 (82.4) |  |
| 300 mg | 0 (0) | 3 (23.1) | 6 (17.6) |  |
| Previous TNFi, n (%) | - | 12 (92.3) | 34 (100) | <0.001 |
| Dose intensification, n (%) | 2 (7) | 2 (15.4) | 7 (20.6) | 0.304 |
| AxSpA: Spondyloarthritis; ASDAS: ankylosing spondylitis disease activity score; BASDAI: Bath ankylosing spondylitis disease activity index; CRP: C-reactive protein; ESR: erythrocyte sedimentation rate; IQR: interquartile range; SD: standard deviation. | | | | |

**Supplementary Table 3.** Sample retention rate during the first year of secukinumab treatment according to diagnosis, gender, and BMI.

| **Diagnosis** | **Retention % (n survival/n total)** | **95%CI** | **Gender** | **Retention % (n survival/n total)** | **95%CI** | **BMI** | **Retention % (n survival/n total)** | **95%CI** |
| --- | --- | --- | --- | --- | --- | --- | --- | --- |
| AxSpA | 78% (61/77) | (73%; 83%) | Female | 95% (19/20) | (89%; 100%) | <30 kg/m2 | 93% (13/14) | (85%; 100%) |
|  |  |  |  |  |  | ≥30 kg/m2 | 100% (6/6) | - |
|  |  |  | Male | 72% (42/57) | (66%; 78%) | <30 kg/m2 | 77% (36/46) | (70%; 84%) |
|  |  |  |  |  |  | ≥30 kg/m2 | 50% (6/11) | (31%; 69%) |
| PsA | 73% (45/61) | (66%; 80%) | Female | 56% (16/28) | (44%; 68%) | <30 kg/m2 | 39% (7/18) | (24%; 54%) |
|  |  |  |  |  |  | ≥30 kg/m2 | 90% (9/10) | (78%; 100%) |
|  |  |  | Male | 88% (29/33) | (81%; 95%) | <30 kg/m2 | 91% (22/24) | (83%; 99%) |
|  |  |  |  |  |  | ≥30 kg/m2 | 78% (7/9) | (63%; 93%) |
| AxSpA: Spondyloarthritis; BMI: Body mass index; 95% CI: 95% Confidence interval; PsA: Psoriatic arthritis. | | | | | | | | |

**2. Supplementary Figure**

**Supplementary Figure 1**

A

B
